# Promoter polymorphisms in the chitinase 3-like 1 gene influence the serum concentration of YKL-40 in Danish patients with rheumatoid arthritis and in healthy subjects

**DOI:** 10.1186/ar3391

**Published:** 2011-06-29

**Authors:** Kaspar R Nielsen, Rudi Steffensen, Martin Boegsted, John Baech, Soeren Lundbye-Christensen, Merete L Hetland, Sophine B Krintel, Hans E Johnsen, Mette Nyegaard, Julia S Johansen

**Affiliations:** 1 Department of Clinical Immunology, Aalborg Hospital, Aarhus University Hospital, Reberbansgade, Pobox 561, 9000, Aalborg, Denmark; 2 Department of Haematology, Aalborg Hospital Science and Innovation Center, Aarhus University Hospital, Soenderskovvej 15, 9000, Aalborg, Denmark; 3 Department of Cardiology, Center for Cardiovascular Research, Aalborg Hospital, Aarhus University Hospital, Soenderskovvej 15, 9000, Aalborg, Denmark; 4 Department of Rheumatology, Copenhagen University Hospital, Hvidovre and Glostrup, Ndr, Ringvej 57, 2600, Glostrup, Denmark; 5 Department of Medicine, Copenhagen University Hospital, Herlev - Ringvej 75, 2730 Herlev, Denmark; 6 Department of Oncology, Copenhagen University Hospital, Herlev - Ringvej 75, 2730 Herlev, Denmark

## Abstract

**Introduction:**

The present study investigates the association between single nucleotide polymorphisms (SNPs) in the chitinase 3-like 1 (*CHI3L1) *gene and serum concentrations of YKL-40 in Danish patients with rheumatoid arthritis (RA) and healthy controls as well as the association with RA in the Danish population. The *CHI3L1 *gene is located on chromosome 1q32.1 and encodes the YKL-40 glycoprotein. YKL-40 concentrations are elevated in the serum of patients with RA compared to healthy subjects, and YKL-40 has been suggested to be an auto-antigen and may play a role in development of RA and in inflammation.

**Methods:**

Eight SNPs in the *CHI3L1 *gene and promotor were genotyped in 308 patients with RA and 605 controls (healthy blood donors) using TaqMan allele discrimination assays. Serum concentrations of YKL-40 were determined by an enzyme-linked immunosorbent assay (ELISA).

**Results:**

We found significant association between the serum concentrations of YKL-40 and polymorphism in the *CHI3L1 *gene among both patients with RA and controls. The g.-131(C > G) polymorphism (rs4950928) was most strongly associated with age adjusted serum concentrations of YKL-40 in patients with RA (*P *< 2.4e-8) and controls (*P *< 2.2e-16). No significant allelic- or genotypic association with RA was found in this Danish cohort.

**Conclusions:**

We suggest that the g.-131(C > G) promoter polymorphism has a substantial impact on serum concentrations of YKL-40 in patients with RA and healthy subjects. However, the polymorphism does not seem to confer risk to RA itself. The effect of *CHI3L1 *polymorphism on clinical outcome or the response to treatment in patients with RA remains to be investigated.

## Introduction

Rheumatoid arthritis (RA) is a systemic autoimmune inflammatory disorder, affecting approximately 1% in western populations. The disease is primarily characterised by chronic polyarthritis [1,2]. The aetiology of RA remains unknown, although it is estimated that the contribution of genetic factors is about 50 to 60% [3,4]. The strongest genetic association is with polymorphic alleles within the human leukocyte antigen HLA-DRB1 locus on chromosome 6p21.3 and a single nucleotide polymorphism (SNP) in the *PTPN22 *gene on chromosome 1p13.2 [5]. Another proposed potential loci is on chromosome 1q32.1 harbouring the chitinase 3-like 1 (*CHI3L1*) gene encoding the YKL-40 protein [6]. YKL-40 is a 40 kDa heparin- and chitin-binding glycoprotein, and a member of chitinase like proteins. YKL-40 is expressed by a variety of cells, including macrophages, neutrophils, synovial cells, arthritic chondrocytes and cancer cells [7-10]. As YKL-40 contains HLA-DR4 binding motifs, it has been suggested to function as an auto antigen in RA [11-15].

A high serum concentration of YKL-40 is emerging as a new biomarker of severe disease activity and poor prognosis in patients with diseases characterized by inflammation and ongoing tissue remodelling such as RA, inflammatory bowel disease, asthma and cancer [8,10,16-26]. The exact biological function of the YKL-40 protein is still largely elusive. YKL-40 is a trans-membrane protein in which cleavaged components bind to an unidentified receptor and the expression of YKL-40 is regulated by various inflammatory cytokines and hormones [27-30]. It is suggested that YKL-40 plays a role in cell proliferation, differentiation and protection against apoptotic signals, and has an effect on extracellular tissue remodelling [31,32]. Two recent studies have explored the effect of YKL-40 as a stimulator of angiogenesis in tumours, suggesting that anti-YKL-40 antibodies could have a place in cancer treatment [33,34].

The proximal promoter region of the *CHI3L1 *gene contains a highly polymorphic area, suggesting a possibility for several functional variants of the gene. Rehli *et al*. [35] demonstrated that binding of the SP1 transcription factor to the most proximal part of the *CHI3L1 *gene affected gene transcription. This finding was supported by Zhao *et al*. [36] reporting functional variants based on the binding of the MYC/MAX transcription factors to the proximal promoter region. The relationships between *CHI3L1 *polymorphisms and YKL-40 production have been studied in a small number of patients with various inflammatory disorders, such as sarcoidosis, asthma, hepatitis, schizophrenia and diabetes [37-44]. These studies suggest that serum concentrations of YKL-40 are, at least partly, regulated by polymorphisms in the proximal promotor region. The findings have been somewhat contradictory and the exact position of the regulatory site or sites remains to be demonstrated. Allele frequencies differ significantly between Caucasian, African and Asian populations, and possibly even within these populations, thereby making direct comparison of the reported studies difficult [45].

Only one small study has evaluated *CHI3L1 *polymorphisms in patients with RA [46]. In 182 Hungarian patients with RA and 194 healthy controls there were no significant differences in genotype frequencies for the g.-131(C > G) or the g.-329(C > T) polymorphisms between the two groups. This study did not evaluate the functional properties of these polymorphisms. Several questions remain unanswered, namely the relationship between *CHI3L1 *polymorphisms and serum concentrations of YKL-40 in patients with RA, the association of *CHI3L1 *promoter genotypes to risk of RA and the Linkage Disequilibrium (LD) properties in different populations.

We aimed to investigate these questions in a cohort of well defined Danish patients with RA and a group of healthy Danish controls. Our hypothesis was that polymorphisms in the proximal promoter region of *CHI3L1*, most likely the g.-131(C > G) polymorphism (rs4950928), are associated with serum concentrations of YKL-40 in both patients with RA and healthy controls. Moreover, we hypothesized that these polymorphisms could be associated with the risk of developing RA and possibly also associated to IgM rheumatoid factor (RF), since YKL-40 seems to play a role in the pathogenesis and immunomodulation in RA.

## Materials and methods

### Patients with rheumatoid arthritis

Three-hundred and eight patients with RA treated at the Department of Rheumatology, Hvidovre Hospital, Hvidovre, Denmark were included in the study. The patients had RA according to the ACR 1987 criteria [47]. The patients with available blood samples were identified in the DANBIO Registry (The Copenhagen Cohort). DANBIO is a Danish nationwide registry that prospectively collects clinical data on patients with rheumatic diseases receiving medical treatment [48]. The blood samples (serum and whole blood) were collected at the time of diagnosis or at the time of starting treatment with TNFα inhibitors. All patients provided informed consents for inclusion in the study population. The study was approved by the local ethics committee. Table 1 summarizes the demographic data for the patients with RA and the controls.

**Table 1:** Characteristics of the study population

	Group			
	**All RA** **(n = 308)**	**IgM RFpos RA** **(n = 178)**	**IgM RFneg RA** **(n = 130)**	**Controls** **(n = 605)**

**Age in years (mean ± SD and range)**	54.5 ± 14.7 (22 to 93)	56.2 ± 14.0 (22 to 86)	52.4 ± 15,4 (23 to 93)	42.6 ± 12.8 (19 to 65)
**Male/Female**	74/234	47/131	27/103	367/238
**Serum YKL-40 ng/ml (median and 95% CI) **	86 (79 to 94)	91 (81 to 102)	80 (70 to 91)	46 (44 to 48)

CI, confidence interval; RA, rheumatoid arthritis; RF, rheumatoid factor.

### Healthy controls

Six-hundred and five healthy blood donors from the Aalborg Hospital Blood Bank, Aalborg, Denmark were included in the study. The donors were known not to take any medication and were clinically healthy at the time of blood drawing. The over-2representation of female controls was a random phenomenon. The samples were handled anonymously and all donors gave consent to the blood being used for this purpose and the sampling was approved by the local ethics committee.

### Handling of blood samples

From the patients with RA and blood donors Ethylenediaminetetraacetic acid (EDTA)-stabilised whole blood and blood samples without anticoagulants were drawn. Serum was isolated from coagulated whole blood within three hours and stored at -80°C until analysis of YKL-40 and IgM-RF was performed. Genomic DNA was prepared from EDTA-stabilised blood samples using a Maxwell 16 blood DNA purification kit (Promega, Madison, WI, USA).

### Biochemical analysis

Serum concentration of YKL-40 was measured by a commercial two-site sandwich type ELISA (Quidel, Mountain View, CA, USA) [49]. The detection limit was 10 ng/ml. The intra-assay coefficient of variations (CV) was 5% and the inter-assay CV was < 6%. IgM-RF was measured using an ELIA fluorescence immunoassay on a Unicap250 system (Phadia AB, Uppsala, Sweden). A validated diagnostic cut off (< 17 kI U/l) was used to classify patients as IgM-RF negative or IgM-RF positive.

### Genotyping

A total of eight SNPs located within the promoter or coding regions of the *CHI3L1 *gene was analysed. Genotyping was performed using real-time polymerase chain reaction (rt-PCR) with TaqMan^® ^SNP Genotyping Assays (Applied Biosystems, Foster City, CA, USA). Applied Biosystems: assay identification numbers are reported as SNP identification. DNA amplification was carried out in a 5 μl volume containing 20 ng DNA, 0.9 μM primers and 0.2 μM probes (final concentrations). The product was amplified using TaqMan Universal PCR Master Mix (Applied Biosystems). Reactions were performed in 384-well plates with the following protocol on a GeneAmp PCR 9700 or a 7900 HT Sequence Detection System: 95°C for 10 minutes, followed by 40 cycles at 95°C for 15 seconds and 60°C for 1 minute. To determine genotypes, end-point fluorescence was read on the 7900 HT Sequence Detection Systems using SDS version 2.3 software (QIAGEN Inc. 27220 Turnberry Lane, CA 91355, USA).

### Statistical analysis

The genotype distribution among patients with RA and controls was tested for deviation from Hardy-Weinberg equilibrium and haplotypes were estimated using the Helix Tree SNP analysis software package (Golden Helix Software, Bozeman, MT, USA). The degree of LD between the SNPs was determined using the SHEsis software (Bio-X Center, Shanghai Jiao Tong University, 1954 Huashan Road, Shanghai 200030, China) [50]. Serum concentrations of YKL-40 were log-normally distributed and, therefore, log-transformed before analysis. Statistical analysis was performed using the statistical software system R, version 2.12.1 [51]. The initial non-linear association between serum concentrations of YKL-40 and age was modelled by a restricted cubic spline function, using the user-contributed package design [52] integrated in R. Analysis of variance based on multiple linear regression models was used to investigate the association between age, gender, case-control status, genotypes and serum YKL-40. Prior to SNP-wise association analysis with serum YKL-40, all serum concentrations of YKL-40 were age adjusted to 44.4 years (mean age for the total sample of controls and cases age 65 years and below) using a linear model. Genotypic associations with age-adjusted serum concentrations of YKL-40 were carried out for cases (age 65 years and below) and controls separately using a multiple linear regression model. For association analysis with RA, allelic and genotypic association was performed using Fisher:s exact test including all patients (n = 308) and controls (n = 605) and using a significance level of 0.05.

## Results

No deviations from Hardy-Weinberg equilibrium were found for any of the eight SNPs in the patient or control group. Age stratification into one-year age groups did not reveal deviations from Hardy-Weinberg equilibrium in any of the age groups.

**Figure 1:**
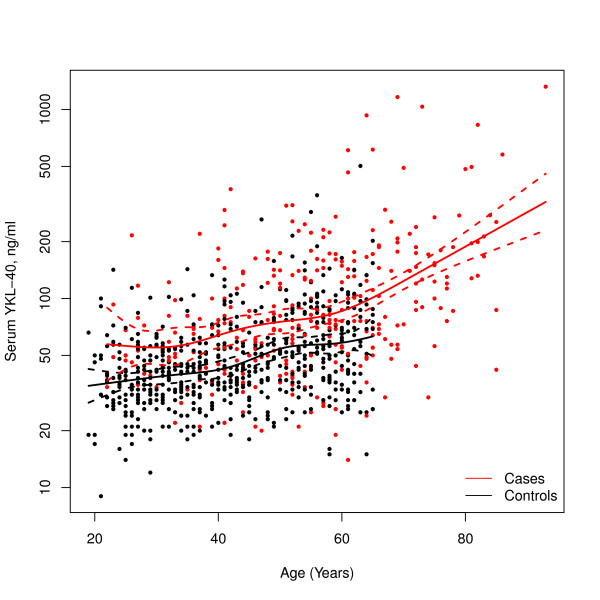
**Non-linear association between age and serum concentrations of YKL-40**. Restricted cubic spline model with six knots applied for patients with rheumatoid arthritis (RA) and controls.

Prior to the SNP association analysis, the effect of age and case-control status on serum YKL-40 was tested using a multiple linear regression model, with serum YKL-40 as dependent variable and case-control status and a non-linear function of age included as covariate. Strong significant association of the serum concentration of YKL-40 with age (*P *< 2.0e-16) and case-control status (*P *< 2.0e-16) was observed (Figure 1). Moreover an apparent increase in serum YKL-40 with age was found for the older patients in the case group. To avoid a potential bias due to the high influence of individuals older than 65 years in the RA group, we excluded in all further analysis patients with RA older than 65 years.

To test the effect of genotypes on serum concentrations of YKL-40 in the RA group (age 65 and below) and control group, a multiple linear regression model including serum YKL-40 as dependent variable and a non-linear function of age, case-control status, genotypes and gender as well as the interaction between case-control status and genotype with age as independent variables was applied (Table 2). From this analysis a strong association was observed with case-control status (*P *< 2.0e-16) (as before), age (*P *< 2.0e-16) (as before) and genotype (*P *< 2.0e-16).

**Table 2:** Sequential analysis of variance table for the regression model for patients ≤ 65 years of age.

Effects	Df	SSQ	F value	Pr(> F)
***Main Effects***				

Age	1	41.844	180.7462	< 2e-16
Status	1	23.888	103.1865	< 2e-16
Genotype	14	51.14	15.7787	< 2e-16
Sex	1	0.516	2.2304	0.1357
***Interactions effects***				
Genotype * Status	13	4.202	1.3961	0.1551
(Status + Genotype) * Age	13	1.671	0.5552	0.8896
(Status + Genotype) * RCS(Age)	55	14.932	1.1727	0.1892
***Error***				
Residual	744	172.24		

RCS, (restricted cubic spline denotes a non-linear relation with age)

**Figure 2:**
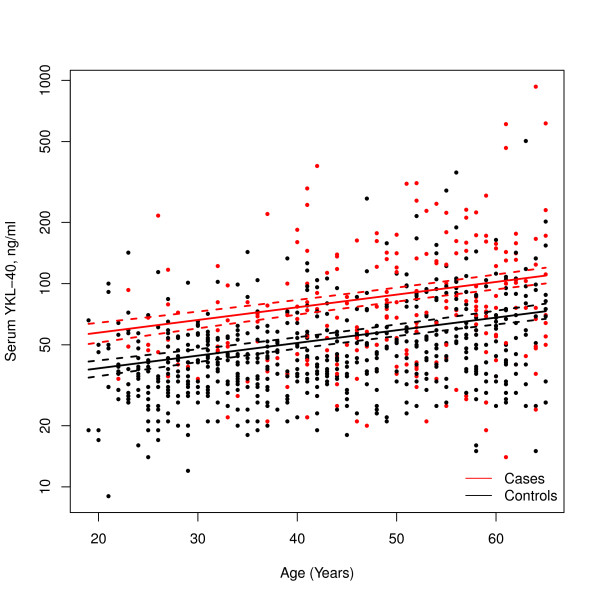
**Linear association between age and serum concentrations of YKL-40**. Linear model applied for patients with rheumatoid arthritis (RA) ≤ 65 years of age (n = 238) and controls (n = 605) is sufficient to explain the age dependent variation (*P *= 0.96). The y-axis represents serum concentrations of YKL-40. Dotted lines represent 95% confidence intervals.

Regarding the age-dependent increase in the serum concentrations of YKL-40, no significant difference was found between a non-linear and a linear model for the age-dependence in both the case group (age 65 and below) and control group (*P *= 0.19) suggesting that the linear model can be used for age adjustment of the serum concentrations of YKL-40 in both groups. The linear model was fitted and depicted in Figure 2.

Serum concentrations of YKL-40 were not associated with gender (*P *= 0.16). There were no interaction effects between case-control status or genotype and age (*P *= 0.89) and no association between serum YKL-40 and the interaction effect between genotype and case-control status (*P *= 0.16) (Table 2). This suggests that age, case-control status and genotypes are all strong independent factors affecting serum concentrations of YKL-40.

**Figure 3:**
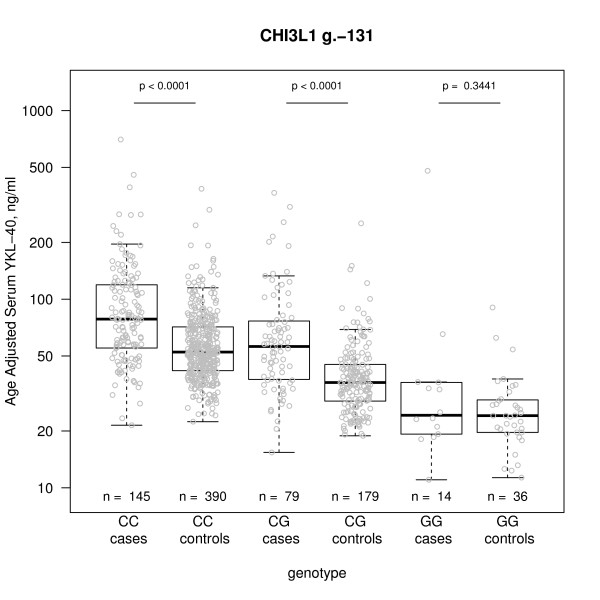
**Association between the *CHI3L1 *g.-131(C > G) polymorphism and age adjusted serum concentrations of YKL-40**. Box plot illustrating the association in 238 patients with rheumatoid arthritis (RA) ≤ 65 years of age (*P *< 2.0e-16 and 605 healthy controls (*P *< 1.1e-8). The x-axis represents *CHI3L1 *g.-131(C > G) genotypes. The y-axis represents serum YKL-40, horizontal bars represents median serum YKL-40 and quartiles for patients with RA and controls.

To test the association of each SNP on age-adjusted serum YKL-40 in the RA group (age 65 and below) and control group, a linear age-adjustment was applied and genotypes were included one-by-one as dependent variables in a multiple linear regression analysis. The g.-131(C > G) genotype was found to be most strongly associated with age-adjusted serum concentrations of YKL-40 in both the patient (*P *= 2.4e-08) and control group (*P *< 2.2e-16) (Table 3). Consistently within both groups, the rare GG genotype was associated with low serum YKL-40, the CG genotype with intermediate serum concentrations of YKL-40, and the common CC genotype with high serum YKL-40 (Figure 3). With respect to genotypes, the RA patients had significantly higher serum YKL-40 than controls for both the CC and CG group. For the rare GG group, the difference was not significant, most likely because of low statistical power due to the limited number of individuals in the GG groups.

**Table 3:** HI3L1 genotypes and the effect on serum YKL-40 levels in patients with rheumatoid arthritis and controls.

SNP	*CHI3L1 *position	Controls (n = 605)	F df = 603	*P*-value	RA age ≤ 65 years (n = 238)	F Df = 236	*P*-value
**rs6691378**	g.-1371G/A	Serum YKL-40 ng/ml			serum YKL-40 ng/ml		
	G/G	46 (44 to 48)	6.63	0.0014	67 (61 to 74)	2.85	**0.06**
	G/A	54 (50 to 59)			77 (62 to 95)		
	A/A	47 (34 to 68)			151 (71 to 323)		
**rs10399931**	**g.-329C/T **						
	C/C	56 (53 to 58)	83.13	< 2.2e-16	80 (71 to 89)	12.34	**8.0e-06**
	C/T	40 (38 to 42)			61 (53 to 70)		
	T/T	25 (22 to 29)			35 (24 to 49)		
**rs10399805**	**g.-247G/A**						
	G/G	45 (44 to 47)	7.47	6.3e-3	67 (61 to 74)	2.69	**0.07**
	G/A	54 (50 to 59)			76 (61 to 94)		
	A/A	48 (34 to 68)			151 (71 to 323)		
**rs4950928**	**g.-131C/G**						
	C/C	56 (53 to 58)	102.32	< 2.2e-16	81 (73 to 90)	18.91	**2.4e-08**
	C/G	38 (38 to 40)			59 (51 to 68)		
	G/G	25 (22 to 28)			31 (22 to 43)		
**rs7515776**	**g.+48A/T**						
	A/A	46 (44 to 48)	7.02	9.7e-3	67 (61 to 74)	1.76	**0.17**
	A/T	54 (50 to 59)			77 (62 to 95)		
	T/T	48 (33 to 70)			119 (62 to 231)		
**rs1538372**	**g.+1219G/A**						
	G/G	56 (53 to 59)	46.93	< 2.2e-16	78 (68 to 89)	7.58	**6.5e-3**
	G/A	43 (41 to 45)			69 (60 to 78)		
	A/A	34 (30 to 37)			45 (34 to 57)		
**rs2071579**	**g.+2117G/C**						
	C/C	56 (52 to 59)	21.91	6.5e-10	72 (62 to 84)	6.31	**0.0022**
	C/G	47 (44 to 49)			76 (67 to 86)		
	G/G	39 (36 to 42)			52 (43 to 62)		
**rs880633**	**g.+2950C/T**						
	C/C	56 (52 to 59)	21.91	6.7e-10	72 (62 to 84)	6.31	**0.0022**
	C/T	47 (44 to 49)			76 (67 to 86)		
	**T/T**	**39 (36 to 42)**			**52 (43 to 62)**		

Serum concentrations of YKL-40 are given as median ± 95% CI.

CHI3L1, chitinase 3-like 1 gene; CI, confidence interval; RA, rheumatoid arthritis; SNP, single nucleotide polymorphism.

When the g.-131 C/G was used as a covariate to determine the influence of the remaining seven SNPs on serum concentrations of YKL-40 none of the other SNPs contributed significantly to the association supporting the isolated highly significant effect of the g.-131 C/G polymorphism on serum concentrations of YKL-40 (Table 4).

**Table 4:** g.-131(C/G) used as a covariate to determine the influence of the remaining 7 SNP:s on s-YKL-40

SNP	*CHI3L1 *position	*P*- value
rs4950928	g.-131C/G	< 2.2e-16
rs6691378	g.-1371G/A	0.21
rs10399931	g.-329C/T	0.88
rs10399805	g.-247G/A	0.19
rs7515776	g.+48A/T	0.25
rs1538372	g.+1219G/A	0.57
rs2071579	g.+2117G/C	0.72
rs880633	g.+2950C/T	0.72

CHI3L1, chitinase 3-like 1 gene; SNP, single nucleotide polymorphism, s-YKL-40 serum concentrations of YKL-40.

**Figure 4:**
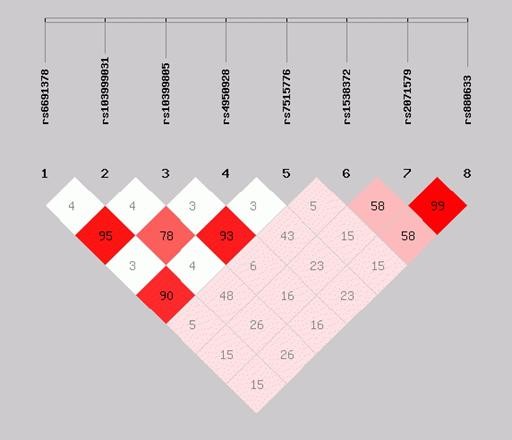
**Linkage disequilibrium in the Danish control individuals (R^2 ^values)**. SNPs are defined by RefSNP number. SNP: single nucleotide polymorphism.

**Figure 5:**
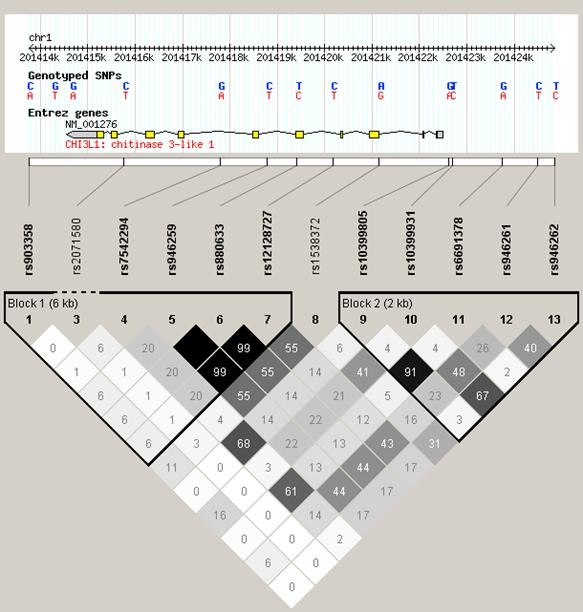
**Linkage disequlibrium between SNPs in the CHI3L1 gene in the CEU HapMap population**. All SNPs are defined by RefSNP number. CHI3L1, chitinase 3-like 1 gene; SNP, single nucleotide polymorphism.

Haplotype analysis did not add further information as all the haplotypes associated with low serum concentrations of YKL-40 carried the g.-131G allele and no further increase in association was seen with any of the haplotypes (data not shown). LD analysis of the eight genotyped SNPs revealed that both the proximal promoter and the distal part of the gene contained blocks of high or moderate LD (Figure 4) explaining the effect of all the included polymorphisms on serum YKL-40 when analysed individually. In particular the -131 C/G polymorphism displayed moderate LD with g.-329C/T (R^2 ^0.78) indicating that the effect on serum concentrations of YKL-40 with g.-329C/T is caused by LD. These findings are in line with CEU HapMap data (Figure 5).

To investigate the association of the eight SNPs with case-control status, allelic and genotypes were tested for association with RA using Fishers exact test. No association was found with alleles or genotypes for any of the eight SNPs (Table 5) indicating that these SNPs do not confer risk to the development of RA itself.

**Table 5:** Association of *CHI3L1 *SNPs with rheumatoid arthritis

SNP	SNP location	*CHI3L1 *position	RA (n = 308)	Controls (n = 605)	*P*-value
**rs6691378**	**Promoter**	**g.-1371G/A**	n (%)	n (%)	
		G/G	249 (80.8)	468 (77.4)	0.48
		G/A	56 (18.2)	130 (21.5)	
		A/A	3 (1.0)	7 (1.2)	
		**Allele**			
		G	554 (89.9)	1,066 (88.1)	0.24
		A	62 (10.1)	144 (11.9)	
**rs10399931**	**Promoter**	**g.-329C/T **			
		C/C	175 (56.8)	360 (59.5)	0.61
		C/T	116 (37.7)	208 (34.4)	
		T/T	17 (5.5)	37 (6.1)	
		**Allele**			
		C	466 (75.6)	928 (76.7)	0.62
		T	150 (24.4)	282 (23.3)	
**rs10399805**	**Promoter**	**g.-247G/A**			
		G/G	248 (80.5)	464 (76.7)	0.38
		G/A	56 (18.2)	134 (22.1)	
		A/A	4 (1.3)	7 (1.2)	
		**Allele**			
		G	552 (89.6)	1,062 (87.8)	0.25
		A	64 (10.4)	148 (12.2)	
**rs4950928**	**Promoter**	**g.-131C/G**			
		C/C	190 (61.7)	390 (64.5)	0.61
		C/G	101 (32.8)	179 (29.5)	
		G/G	17 (5.5)	36 (6.0)	
		**Allele**			
		C	481 (78.1)	959 (79.3)	0.56
		G	135 (21.9)	251 (20.7)	
**rs7515776**	**Intron 1/ exon 1**	**g.+48A/T**			
		A/A	247 (80.2)	467 (77.2)	0.20
		A/T	55 (17.9)	132 (21.8)	
		T/T	6 (1.9)	6 (1.0)	
		**Allele**			
		A	549 (89.1)	1,066 (88.1)	0.52
		T	67 (10.9)	144 (11.9)	
**rs1538372**	**Intron 2/ exon 3**	**g.+1219G/A**			
		G/G	137 (44.5)	277 (45.8)	0.93
		G/A	137 (44.5)	262 (43.3)	
		A/A	34 (11.0)	66 (10.9)	
		**Allele**			
		G	411 (66.7)	816 (67.4)	0.76
		A	205 (33.3)	394 (32.6)	
**rs2071579**	**intron 4/ exon 4**	**g.+2117G/C**			
		C/C	63 (20.5)	123 (20.3)	0.89
		C/G	149 (48.4)	302 (49.9)	
		G/G	96 (31.2)	180 (29.8)	
		**Allele**			
		C	275 (44.6)	548 (45.3)	0.79
		G	341 (55.4)	662 (54.7)	
**rs880633**	**exon 5**	**g.+2950C/T**			
		C/C	96 (31.2)	180 (29.8)	0.88
		C/T	149 (48.4)	303 (50.0)	
		T/T	63 (20.4)	122 (20.2)	
		**Allele**			
		C	341 (55.4)	663 (54.8)	0.82
		T	275 (44.6)	547 (45.2)	

CHI3L1, chitinase 3-like 1 gene; RA, rheumatoid arthritis; SNP, single nucleotide polymorphism.

The high producer genotypes were not more frequent in the IgM-RF positive subgroup and no difference was found in geno- or phenotype distribution between seropositive and seronegative patients with RA (data not shown).

## Discussion

This study aimed to investigate eight polymorphic sites in the *CHI3L1 *gene with possible functional properties in both patients with RA and healthy individuals. We focused on the g.-131(C > G) allele and closely related polymorphisms described in Caucasian populations [26,36-39,43,44,46]. The g.1219(G > A) polymorphism was also included as one study reported an individual functional property of this polymorphism [43]. Serum concentrations of YKL-40 were strongly associated with age and case-control status. After adjustment of the serum concentrations of YKL-40 for these two variables, serum YKL-40 was found to be significantly associated with SNPs in the *CHI3L1 *gene. The strongest association was with the g.-131(C > G) promoter polymorphism. The association of serum YKL-40 with the remaining SNPs could be explained by LD. Our findings indicate that serum concentrations of YKL-40 are under the influence of genetic variability in the *CHI3L1 *gene in both patients with RA and healthy controls, and the effect of genotypes seems to be the same in both groups. Though our results indicate that *CHI3L1 *polymorphisms are not involved in the pathogenesis of RA, we do not know if high producer genotypes results in a more severe clinical phenotype.

Several other studies have suggested the g.-131(C > G) is a strong candidate for a functional promoter polymorphism influencing the serum concentrations of YKL-40 [36,42-45]. The promoter SNP g.-131(C > G) in the *CHI3L1 *gene was associated with elevated serum YKL-40, asthma, bronchial hyper responsiveness and pulmonary function [44,45], and with elevated serum YKL-40 and the severity of hepatitis C virus-induced liver fibrosis [43]. This indicates a functional role of YKL-40 in these diseases. An association is also found between schizophrenia and haplotypes within the promoter region of the *CHI3L1 *gene suggesting that polymorphisms in an area starting from base pair position -180 could have functional properties [36,42]. Our findings support these earlier studies.

Zhao *et al*. [36] investigated Chinese patients with schizophrenia and found lower activity of the transcription factor MYC/MAX and decreased *CHI3L1 *gene expression related to the low frequency G allele for the g.-131(C > G) SNP. Ober *et al*. [44] studied 443 patients with asthma and 491 healthy controls from a genetically preserved group of Americans of European descent. They found that serum concentrations of YKL-40 were associated with many alleles in the promoter region including the g.-131(C > G) and g.-329(C < T) polymorphisms. This supports the g.-131(C > G) polymorphism as a site of genetic regulation in both healthy controls and patients with asthma. Conclusions were complicated by strong LD in the promoter region in the population studied. They also showed a strong association to the g.-1219(G > A) polymorphisms, which was not in LD with the promoter polymorphisms. This individual effect on serum YKL-40 with g.-1219(G > A) was not supported in our study as we found this phenomena related to LD in the Danish population. In contrast, Sohn *et al*. [40] demonstrated a functional effect of the g.-247 (G > A) polymorphisms in a study of 295 atopic children and 180 healthy controls from a Korean population. They concluded that this polymorphism was responsible for most of the genetic effects on YKL-40 production, and that the g.-131C allele was associated to low promoter activity. These results are complicated by the fact that the g.-131(C > G) and g.-247(G > A) polymorphisms showed no LD in the Asian populations, contradictory to our finding which suggest a high degree of LD in this part of the proximal promoter. In the Danish population the region on chromosome 1 bearing the g.-131(C > G) polymorphism was in strong LD, illustrated by the occurrence of just 8 frequent haplotypes (f > 1%). The g.-131(G > C) allele was found to be in LD with several other loci in the *CHI3L1 *gene, and three haplotypes could be defined as low producer haplotypes, all including the g.-131G allele. It must be emphasized that ethnicity seems to play an important role in the genetic regulation of YKL-40 production, and results from our and similar studies can only be considered valid in ethnically similar populations. Further studies in different populations are awaited.

Serum concentrations of YKL-40 increased with increasing age in both healthy controls and patients with RA making age a possible confounding variable. The cause of this remains unknown, but the phenomena has been explained by a higher level of general inflammation and apoptosis in the elderly, which is well known for other inflammatory mediators [53]. Similar increases in plasma YKL-40 with age have recently been described in a large group of 8,899 subjects from the general Danish population [20]. We initially decided to fit a non-linear model to explain the effect of age on serum concentrations of YKL-40. Our control group did not include any persons above the age of 65, but below this age we were able to fit a linear model explaining the relationship between age and serum concentrations of YKL-40. This supports the findings by Kruit *et al*. [38], who suggested a linear relationship between age and serum YKL-40. In the patients with RA it seems as if serum concentrations of YKL-40 rise more rapidly with age above age 65, indicating that elevated serum YKL-40 in this age group needs careful interpretation. It is possible that high serum YKL-40 is associated to co-morbidity or a latent malignant disease [10,20-26,53,54]

It remains unknown whether high serum YKL-40 affects a person:s risk of autoimmune disease in the long term. YKL-40 expression is stimulated by the inflammatory cytokines TNF-α, IL-6 [30] and IL-1β, whereas YKL-40 inhibits cellular responses induced by IL-1 and TNF-α, suggesting an autocrine feed-back mechanism [9,28]. YKL-40 is strongly expressed by macrophages in the synovial membrane of RA patients possibly activated by a pro-inflammatory IFNγ-mediated immune response, and elevated YKL-40 can stimulate local production of anti-inflammatory IL-10 [32]. In inflammatory diseases such as RA, the excessive YKL-40 production may also have the opposite effect stimulating a continuous pro-inflammatory state and stimulation of VEGF and angiogenesis [32-34].

## Conclusions

In conclusion, this study reports a strong association between the g.-131(C > G) allele and serum concentrations of YKL-40 in both patients with RA and healthy controls. Our findings indicate that the g.-131(C > G) polymorphism is the main contributor to the inter-individual variation of serum YKL-40 in Caucasian patients with RA, and that the effect of other polymorphic sites in this region is related to a high degree of LD in this area of the genome.

## Abbreviations

*CHI3L1: *chitinase 3-like 1 gene; CV: coefficient of variations; LD: linkage disequilibrium; RA: rheumatoid arthritis; RF: IgM rheumatoid factor; RS: RefSNP number; RT-PCR: real-time polymerase chain reaction; SNP: single nucleotide polymorphism

## Competing interests

The authors declare that they have no competing interests.

## Authors' contributions

KRN was involved in all aspects of study conception, design, analysis, interpretation and report generation and provided final approval of the version of the submitted manuscript. RS, JB, SLC, MLH, SK, HEJ and JSJ were involved in data acquisition, analysis and report drafting and provided final approval of the submitted manuscript. MB and MN were involved in statistical analysis and linkage analysis and provided final approval of the submitted manuscript.
